# The Effects of Acid Etching on the Nanomorphological Surface Characteristics and Activation Energy of Titanium Medical Materials

**DOI:** 10.3390/ma10101164

**Published:** 2017-10-11

**Authors:** Kuo-Yung Hung, Yi-Chih Lin, Hui-Ping Feng

**Affiliations:** Department of Mechanical Engineering, Ming Chi University of Technology, 84 Gungjuan Rd., Taishan Dist., New Taipei City 24301, Taiwan; haskelhung@gmail.com (Y.-C.L.); hpfeng@mail.mcut.edu.tw (H.-P.F.)

**Keywords:** dental implant, activation energy, sandblasted with large grit and acid-etched (SLA), initial healing, rapid osseointegration, osteoblasts, osteoclasts

## Abstract

The purpose of this study was to characterize the etching mechanism, namely, the etching rate and the activation energy, of a titanium dental implant in concentrated acid and to construct the relation between the activation energy and the nanoscale surface topographies. A commercially-pure titanium (CP Ti) and Ti-6Al-4V ELI surface were tested by shot blasting (pressure, grain size, blasting distance, blasting angle, and time) and acid etching to study its topographical, weight loss, surface roughness, and activation energy. An Arrhenius equation was applied to derive the activation energy for the dissolution of CP Ti/Ti-6Al-4V ELI in sulfuric acid (H_2_SO_4_) and hydrochloric acid (HCl) at different temperatures. In addition, white-light interferometry was applied to measure the surface nanomorphology of the implant to obtain 2D or 3D roughness parameters (Sa, Sq, and St). The nanopore size that formed after etching was approximately 100–500 nm. The surface roughness of CP Ti and Ti-6Al-4V ELI decreased as the activation energy decreased but weight loss increased. Ti-6Al-4V ELI has a higher level of activation energy than Ti in HCl, which results in lower surface roughness after acid etching. This study also indicates that etching using a concentrated hydrochloric acid provided superior surface modification effects in titanium compared with H_2_SO_4_.

## 1. Introduction

The world’s largest dental implant markets are in Europe and the United States. According to Global Markets Direct’s estimates, the U.S. dental implant market in 2001 was approximately US$1.4 billion; in 2008, it had grown to $3.9 billion, which constitutes a compound annual growth rate of approximately 8.4%. It is estimated that by 2025, the market demand may reach approximately $17 billion [1]. Commercially-pure titanium (CP Ti) has been used as a dental implant material for the past 40 years because it is anti-corrosive, exhibits suitable mechanical properties, and has biocompatibility [2]. Fast osseointegration after implantation and strong new bone formation on the implant surface during the healing process constitute high biocompatibility. To achieve a high level of success in clinical surgery, the surface quality and topography of CP Ti are among the most critical factors influencing the results of implant surgery. A substantial majority of the developed and investigated implant surfaces, such as SLA (sandblasted with large grit and acid-etched), are smoother than the turning surfaces. Straumann AG proposed an SLA patent in 1992 [3]. The implant surface is sandblasted using large grit and etched in a boiling mixture of hydrochloric acid or sulfuric acid. The main novel characteristic of this patent is the surface features of Ra = 1–2 μm, Rt = 20–30 μm, and Rs = 1–5 μm. The surface is not microporous and, therefore, provides no enclosed volumes, which reduces vulnerability to bacteria. In recent years, it has become a mainstream and commercially available implant product. Ti implant surfaces have been modified using multiple methods (additive or subtractive processes) such as (additive) hydroxylapatite coating, calcium phosphate coating, Ti plasma spraying, ion deposition, (subtractive) electropolishing, mechanical polishing, blasting, etching, and oxidation. These different techniques are employed to both smoothen and roughen the implant surfaces. Regarding bone integration, smoother surfaces, such as electrically- or mechanically-polished surfaces, may be excessively smooth for adequate osseointegration; however, they may still be used for research purposes if an investigation of certain surface properties is required. Spark erosion techniques can produce a relatively rough surface, but this method is unstable and does not guarantee bone integration, possibly because of the impurities that are incorporated into the surface [4]. Wennerberg [5] discussed the possible relationships between Ti surface topography and the bone integration found in numerous studies. Bone integration has been reported in the results of numerous experiments, and bone response has been reported as influenced by implant surface topography.

Gintaras et al. [6] proposed using a different surface treatment for metals—HCl etching—which did not produce pores on the metal surface. Although HCl/H_2_SO_4_ etching generated sporadic holes, the holes were not obvious. When an H_2_SO_4_/HCl/H_3_PO_4_-etched surface displayed numerous holes, they were undulating and uneven. Gintaras et al. proved that a uniform surface can be fabricated after etching Ti in H_2_SO_4_ for 72 h or through HCl etching for 30 h. However, they did not specify the etchant concentration. Szmukler-Moncler et al. (2003) [7] investigated the surface characteristics of four implant systems: osseotite (3i) (etching in 15% hydrogen fluoride (HF) and a mixture of H_2_SO_4_ and HCl (6:1) at 60–80 °C for 3–10 min), SLA (Straumann, Basel, Switzerland), DPS (Friatec, Mannheim, Germany; DPS implant is processed using sand-blasting and acid-etching), and HaTi (HaTi Dental, Solothurn, Switzerland). The roughness values of the four implant types were measured and ranked in the following order: SLA (Straumann) > DPS (Friatech) > HaTi (HaTi Dental) > osseotite (3i). Le Guehennec et al. [8] reported that numerous holes could be etched by using a mixture of HCl and H_2_SO_4_ at a temperature exceeding 100 °C to the surface of a substrate; this enhanced the adhesion of osteoblasts and fibrin. Ferguson et al. [9] proposed that the difference between SLA and SLA using hydrophilic treatment (SLActive) is the hydrophilicity implant. SLActive displayed superior performance relating to biocompatibility, cell activity, healing time, and mechanical properties compared with SLA. Ban et al. [10] claimed that the surface roughness of CP Ti was strongly related to acid temperature, etching time (0.25–8.00 h), weight loss, and its activation energy in concentrated sulfuric acid (48%).

Surface modification technology, combined with Ti or Ti alloys, can retain the original corrosion resistance and tensile strength of Ti or the Ti alloys and, thus, improve the clinical benefits of implants. However, the existing research on the detailed parameters of the etching recipe, concentration, temperature, and time is limited, and the Ti metal etching methodologies that have been proposed are substantially different. Therefore, the purpose of this paper is to explore the process parameters of the surface treatment, such as the etchant used, acid concentration, reaction temperature, and reaction time, the optimal values of which can be used to obtain uniform nanopores suitable for bone cell growth. In addition, some uncertainties regarding these parameters were addressed, and unified biocompatibility mechanisms of CP Ti acid etching are proposed.

## 2. Materials and Methods

**Figure 1:**
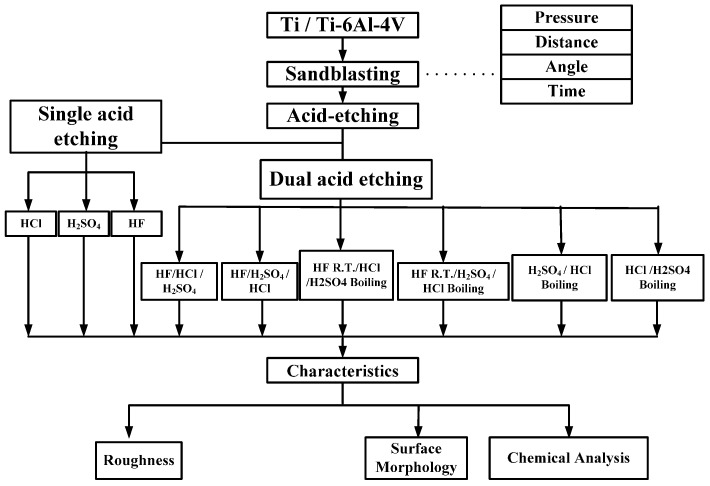
The experimental procedure.

Circular CP Ti and Ti alloy disks (Grade 5 Ti-6Al-4V, ASTM-F136; 0.375″ in diameter and 2 mm thick) cut from a circular bar using wire electrical discharge machining were used to verify the surface characteristics and in etching experiments in this study. Sandblasting and magnetic grinding were performed to clean the disk surface after the wire electrical discharge machining. The experimental procedure is represented in Figure 1. The CP Ti and Ti alloy specimens were sandblasted to roughen the surface and were then cleaned using ultrasonic oscillation. Various etching mechanisms were applied to form a uniform surface with micro- to nano-sized holes. Finally, the results were analyzed to investigate the influences of the process parameters on the roughness, surface morphology, and chemical composition of the materials. The specimens were treated and examined using the following equipment: a sandblasting machine (quad disc blasting machine CS-1000RT, Zhaoshun, Taipei, Taiwan), etching equipment, and an ultrasonic oscillator (Delta Ultrasonic, DC300H, New Taipei City, Taiwan, frequency: 40 kHz). The chemical composition of the specimens was investigated using X-ray diffractometry (X-ray diffractometer, XRD, X’Pert Pro, PHILIPS, Eindhoven, The Netherlands; Cu-K_P Å, 40 kV, 30 mA, scan speed 0.1 s/step). The surface morphologies of the disks were observed using a scanning electron microscope (field emission scanning electron microscope, FE-SEM, JSM-6701F, JEOL, Tokyo, Japan). The surface roughness of the dried specimens was analyzed using a white-light interferometer (Chroma 7502, New Taipei City, Taiwan, vertical resolution: 1 nm, Sa measurement area: 730 × 730 μm^2^). Precision microbalances (RADWAG, MYA 21, Radom, Poland) were used to measure the weight lost by the materials, which was derived from measuring the weight difference before and after the etching was performed.

In this study, the blasting parameters, namely, pressure (A), blasting time (B), blasting distance (C), blasting angle (D), and grain size (E), were tested to assess the surface roughness and characteristics of the implant. Then, the predominant characteristics of Ti or Ti alloy after monoacid etching were determined by calculating the etching rate and observing the surface morphology. In this study, HF, H_2_SO_4_, and HCl were first tested at different temperatures. Whereas Ti has a strong affinity reaction with oxygen, nitrogen, and hydrogen at a high temperature, only the hydrogen absorption is reversible. The Ti neither reacts with cold mineral acids (HF is the exception) nor with a hot alkali solution. However, it can be dissolved in hot HF, HCl, H_2_SO_4_, and H_3_PO_4_. In general, the dissolution accelerates as the acid concentration increases.

### 2.1. Sandblasting

Alumina (Al_2_O_3_) was used as a blasting material and produced various levels of surface roughness according to different parameters: pressure at 2, 3, 4, 5, and 6 kg/cm^2^; distances of 10, 30, 60, 90, and 120 mm; periods of 30, 45, 60, 90, and 120 s; angles at 45° and 90°; and grain sizes of #80 (180–212 µm) and #100 (120–150 µm).

### 2.2. Acid Etching

The etching (acid etching) process is often used to remove the surface oxide layer and the contaminants of Ti to obtain a clean and uniform surface. The etchant of Ti is often dissolved in 10% to 30% nitric acid (HNO_3_) and in 1% to 3% HF in water. Hydrogen fluoride reacts readily with Ti and forms Ti fluoride and hydrogen gas. To prevent hydrogen embrittlement [11], a 10:1 mixture of nitric acid and hydrogen fluoride were normally used to reduce hydrogen generation.

The chemical reaction of Ti and HF is as follows:

$$6HF+Ti\to H_{2}TiF_{6}+2H_{2}↑$$

H_2_SO_4_ oxidizes if its concentration exceeds 85% at any temperature. The reactions of Ti in concentrated H_2_SO_4_ are the following [10]:

$$TiO_{2}+2H_{2}SO_{4}\to Ti{\left(SO_{4}\right)}_{2}+2H_{2}O$$

$$Ti+2H_{2}SO_{4}\to Ti{\left(SO_{4}\right)}_{2}+2H_{2}$$

$$Ti+H_{2}\to TiH_{2}$$

When Titanium reacts with HCl [12], possible reaction are:

$$2Ti+6HCl\to 2TiCl_{3}+3H_{2}$$

$$TiO_{2}+4HCl\to TiCl_{4}+2H_{2}O$$

$$Ti+H_{2}\to TiH_{2}$$

The initial period, in which no weight changes were observed, might represent the time required to dissolve the passive oxide film and expose the metallic Ti to the acid. Table 1 shows the concentration of the acid used for the study.

Table 2 shows the etching parameters for a single type of acid at different temperature. Table 3 shows the etching parameters for multiple types of acid at boiling. According to the weight loss, etching time, and temperature during acid etching, the activation energy can be estimated for the dissolution of CP Ti or Ti6Al4V in a different etchant solution by using the Arrhenius theory. The surface roughness was also measured after the etching process.

The Arrhenius equation [12] is:

$$k=Ae^{\frac{-E_{a}}{RT}}$$

where *R* is the universal gas constant (8.314 J/mol K) and *k* is the number of collisions that result in a reaction per second. *A* is the total number of collisions (both leading to and not leading to a reaction) per second and $e^{\frac{-E_{a}}{RT}}$ is the probability that any given collision results in a reaction. *T* is the temperature in absolute kelvin and *E_a_* (kilojoules per mole) is the activation energy. In the Arrhenius equation, the activation energy is defined as the minimum energy required to initiate the chemical reaction.

Taking the natural logarithm of the Arrhenius equation yields:

$$\ln\left(k\right)=\frac{-E_{a}}{R}\frac{1}{T}+\ln\left(A\right)$$

This has the same form as the equation for a straight line, namely, y=ax+b. Therefore, when a reaction has a rate constant that is consistent with the Arrhenius equation, a plot of ln(*k*) versus *T*^−1^ generates a straight line, the gradient and intercept of which can be used to determine *E_a_* and *A*. That is, the activation energy is defined as (−*R*) times the slope of a plot of ln(*k*) vs. (1/*T*):

$$E_{a}=-R\times \frac{\partial \ln k}{\partial \left(1/T\right)}$$

## 3. Results

### 3.1. Results of Sandblasting

**Figure 2:**
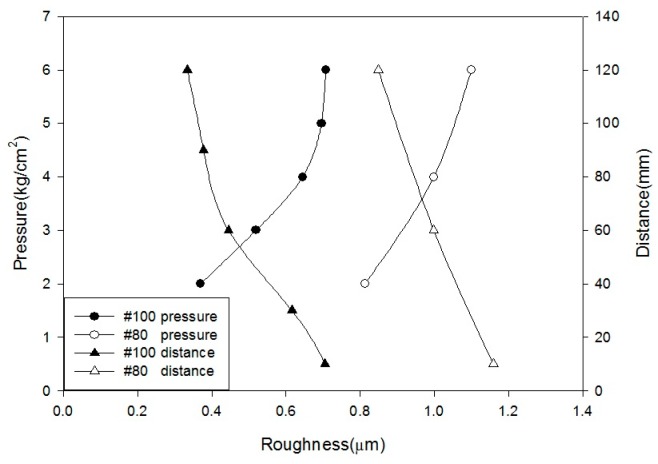
Relation between surface roughness and sandblasting pressure, distance.

**Figure 3:**
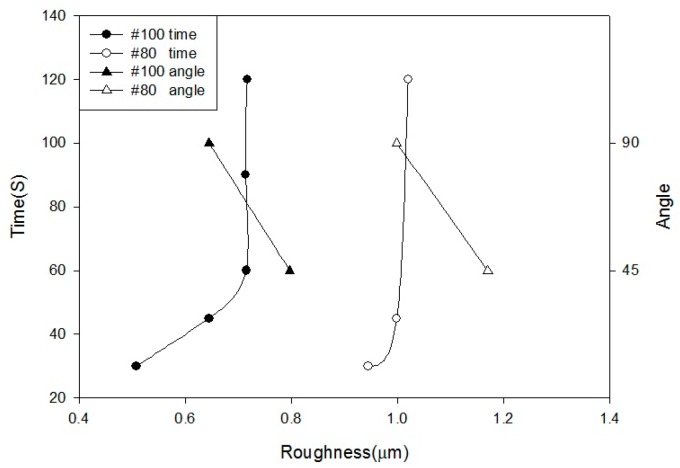
Relation between surface roughness and sandblasting time, angle.

Based on the blasting parameters used in this paper, we have summarized the surface roughness results, as shown in Figure 2 and Figure 3. Figure 2 shows that, as the pressure increases, the changes in the surface roughness become alleviation. When the pressure exceeded 4 kg/cm^2^ (#100), the surface roughness of the specimens remained almost constant. The surface roughness increased as the sandblasting distance decreased. However, the sandblasting distance of 10 mm is considerably close, causing difficulty in obtaining a uniform surface. Figure 3 shows that surface roughness increased when the blasting time increased. After 60 s, the changes become gentler. In addition, the surface roughness of a sandblasting angle at 45° exceeds 90° because it consists of both slamming and cutting forces. Thus, the surface of a sandblasting angle at 45° is substantially rougher than that of a sandblasting angle at 90°. The larger sandblasting size of #80 results in a substantially rougher surface, which is consistent with the sandblasting theory.

### 3.2. Acid Etching

The holes caused by blasting consist of sizeable pores (from 10 μm to approximately 30 μm), and adding an acid etching process could produce sub-micron holes (<1 μm, small holes in the large holes) with specific surface roughness and uniform nanopores on implants. This helps to increase the contact area of the bone for an implant and to reduce the healing time of the implant surgery.

#### 3.2.1. Characteristic Evaluation after Acid Etching

**Figure 4:**
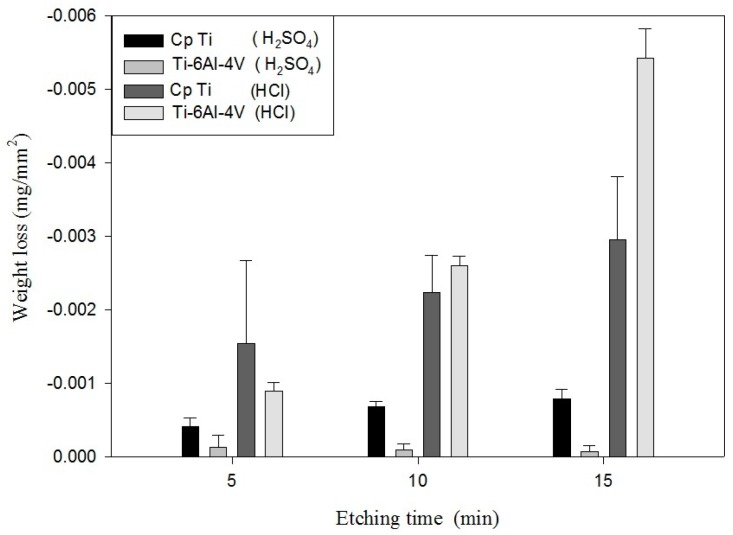
The comparison of the weight loss of Ti/Ti-6Al-4V ELI for different etchant solutions after etching at RT.

**Figure 5:**
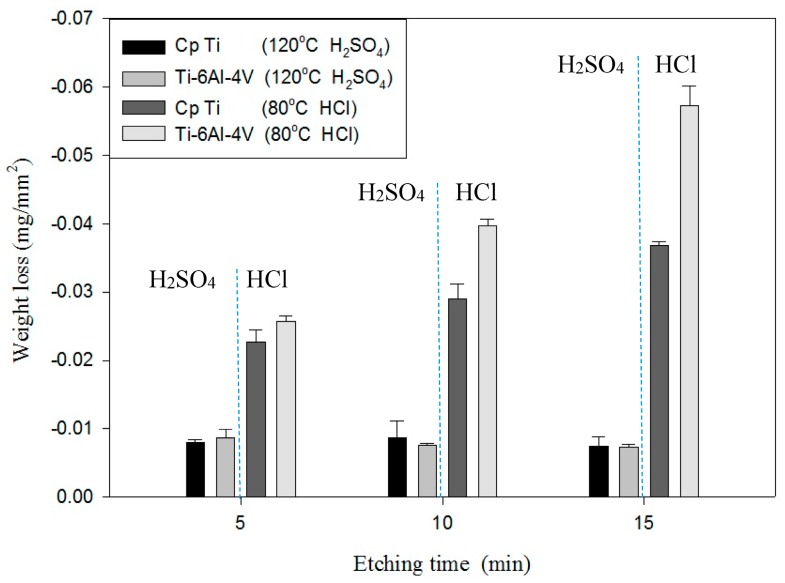
The comparison of the weight loss of Ti/Ti-6Al-4V ELI for different etchant solutions after etching at BT.

In this paper, to understand the etching rate of various acids on Ti/Ti-6Al-4V ELI, the weight loss was measured for 10 min to obtain the etching rates of Ti/Ti-6Al-4V ELI at different temperatures and when using different acid etchants. Table 4 and Table 5 show the etching rates of Ti/Ti-6Al-4V ELI for different etchant solutions at RT or BT. The etching rate of HF at RT for 10 min was 9.28 mg/min. The etching rate of HCl for Ti-6Al-4V was 0.078 mg/min at RT and 0.834 mg/min at BT, and the H_2_SO_4_ at BT for Ti-6Al-4V was 0.009 mg/min at RT and 0.162 mg/min at BT. The etching rate of HCl at boiling for Ti was 0.26 mg/min at RT, 0.659 mg/min at BT, and the H_2_SO_4_ at BT for Ti was 0.005 mg/min at RT and 0.06 mg/min at BT. At RT, the etching rate of HCl and H_2_SO_4_ was extremely slow. Figure 4 and Figure 5 show the comparison of the weight loss of Ti/ Ti-6Al-4V ELI for different etchant solutions and at different temperatures. As the Figure 5 shows, Ti-6Al-4V ELI exhibited poor etching resistance at a BT. Table 6 shows that the difference in weight loss between etching with HF and etching without HF was only 0.01 g. The pre-treatment of the oxide layer of Ti by using HF does not increase the etching rate of Ti, showing that no obvious TiO_2_ layer developed on Ti if the etching process proceeded shortly after blasting because TiO_2_ can be dissolved in HF, concentrated HCl, and hot, concentrated H_2_SO_4_. Therefore, this might be caused by the poor anti-etching ability of TiO_2_ in HCl.

#### 3.2.2. Surface Characteristics

**Figure 6:**
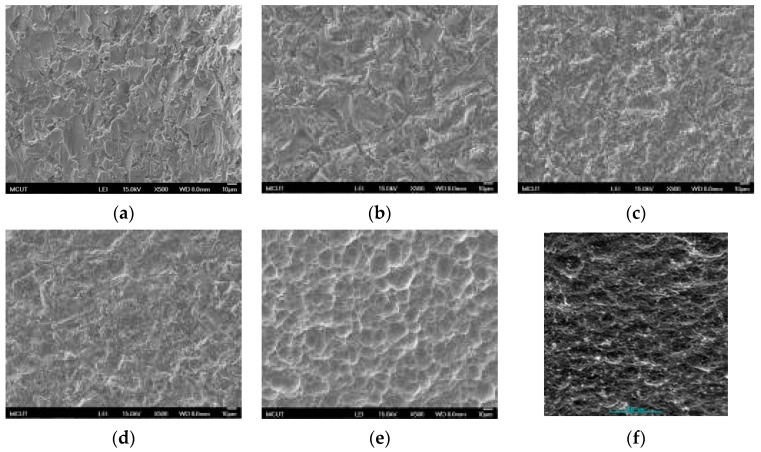
SEM images of Ti-6Al-4V surface after a single acid etching at RT and at BT (500× magnification): (**a**) sandblasting; (**b**) H_2_SO_4_ for 10 min; (**c**) HCl for 10 min; (**d**) H_2_SO_4_ (120 °C) for 10 min; (**e**) HCl (80 °C) for 10 min; and (**f**) HF for 10 min.

**Figure 7:**
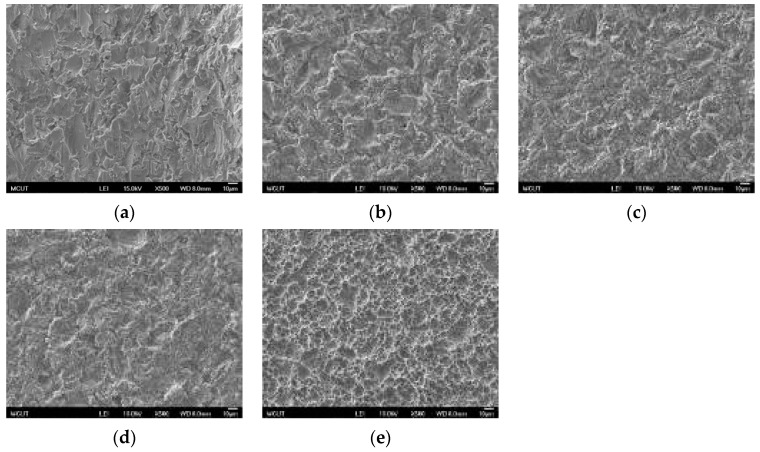
SEM images of CP Ti surface after a single acid etching at RT and at BT (500× magnification): (**a**) sandblasting; (**b**) H_2_SO_4_ for 10 min; (**c**) HCl for 10 min; (**d**) H_2_SO_4_ (120 °C) for 10 min; and (**e**) HCl (80 °C) for 10 min.

Figure 6 shows SEM images of Ti-6Al-4V surface after a single acid etching (H_2_SO_4_, HCl, and HF) at RT and at BT (500× magnification). Figure 6a,b show similar surface characteristics between sandblasting and H_2_SO_4_ etching at RT after sandblasting, proving that H_2_SO_4_ has a low etching rate at RT. Comparing to Figure 6b,c indicates that a porous structure formed after etching in HCl or H_2_SO_4_ at RT. However, the etching rate was considerably low in these two cases. Comparing to Figure 6d,e indicates that porous structures become more obvious after etching in HCl or H_2_SO_4_ at BT. Figure 6f shows the SEM (500×) images of Ti-6Al-4V after HF etching (30 s) at RT. This shows that a high etching rate is unhelpful in forming nanoholes. Since Ti-6Al-4V is an α + β crystal phase material and the etching rate differs for Ti, Al, and V, the variability of the surface roughness was more substantial than that for Ti (Figure 7).

Similar results are illustrated in Figure 7 for Ti etching by using a single acid. However, because of the pureness of the material, the etching rate of the CP Ti surface was the same, resulting in a uniform Ti surface.

**Figure 8:**
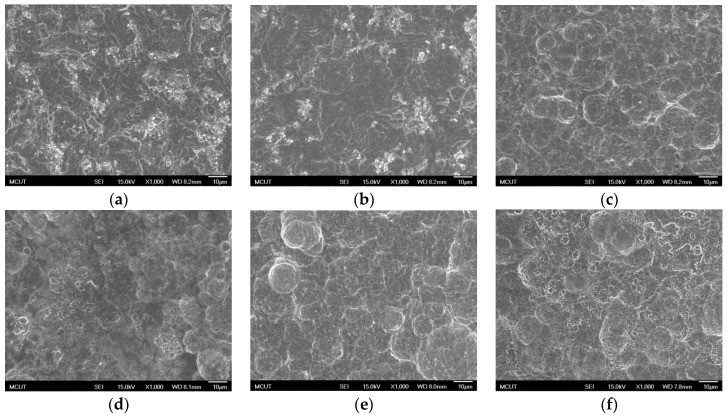
SEM images of Ti-6Al-4V surface after dual acid etching at RT and at BT (1000× magnification): (**a**) HF/H_2_SO_4_/HCl; (**b**) HF/HCl/H_2_SO_4_; (**c**) HF/H_2_SO_4_ (120 °C)/HCl (80 °C); (**d**) HF/HCl (80 °C)/H_2_SO_4_ (120 °C); (**e**) H_2_SO_4_ (120 °C)/HCl (80 °C); and (**f**) HCl (80 °C)/H_2_SO_4_ (120 °C).

Figure 8 shows SEM images of the Ti-6Al-4V surface after dual acid etching at RT and at BT. Figure 8e,f show that using the same two types of etchant, but in a different order, resulted in a completely different surface morphology. Numerous micrometer (20–30 μm) and nanometer holes were formed after etching by using H_2_SO_4_/HCl, (Figure 8e).

**Figure 9:**
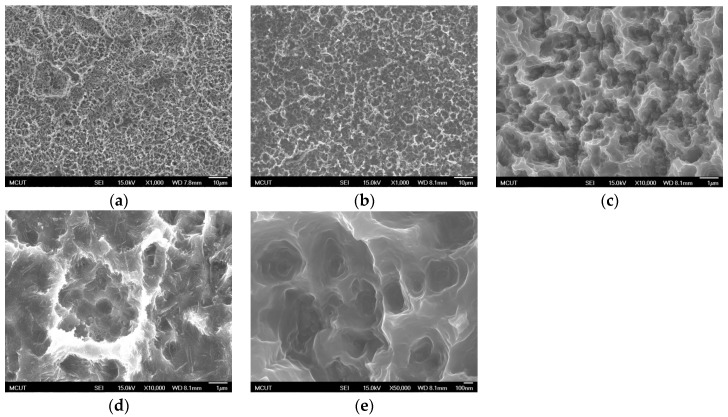
SEM images of CP Ti surface after dual acid etching at BT for 15 min: (**a**) H_2_SO_4_ (120 °C)/HCl (80 °C) (1000× magnification); (**b**) HCl (80 °C)/H_2_SO_4_ (120 °C) (1000× magnification); (**c**) H_2_SO_4_ (120 °C)/HCl (80 °C) (10,000× magnification); (**d**) HCl (80 °C)/H_2_SO_4_ (120 °C) (10,000× magnification); and (**e**) H_2_SO_4_ (120 °C)/HCl (80 °C) (50,000× magnification).

Figure 9a–d shows the SEM images (1000× and 10,000× magnification) of CP Ti surface after dual acid etching (H_2_SO_4_/HCl and HCl/H_2_SO_4_) at BT, demonstrating that etching in a H_2_SO_4_/HCl order could produce a more uniform surface morphology and nano-pore structure than could be produced using a HCl/H_2_SO_4_ order. Figure 9e is an SEM image with 50,000× magnification, which shows that numerous uniform nanometer holes were obtained after acid etching. Since these holes have a considerably deep spiral structure and uniform surface, they help the osteoblast tentacles reach deeply and increase the stability of the implant after implantation.

**Figure 10:**
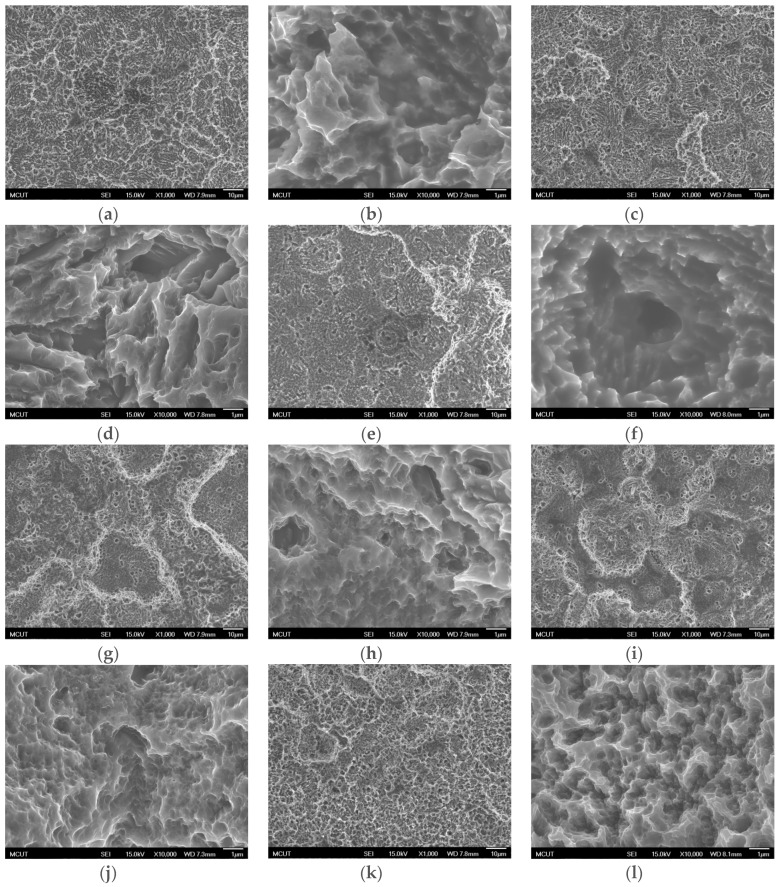
SEM images of the CP Ti surface etching in H_2_SO_4_/HCl at different temperatures. (**a**) H_2_SO_4_ (90 °C)/HCl (80 °C) (1000× magnification); (**b**) H_2_SO_4_ (90 °C)/HCl (80 °C) (10,000× magnification); (**c**) H_2_SO_4_ (100 °C)/HCl (80 °C) (1000× magnification); (**d**) H_2_SO_4_ (90 °C)/HCl (80 °C) (10,000× magnification); (**e**) H_2_SO_4_ (110 °C)/HCl (80 °C) (1000× magnification); (**f**) H_2_SO_4_ (110 °C)/HCl (80 °C) (10,000× magnification); (**g**) H_2_SO_4_ (120 °C)/HCl (70 °C) (1000× magnification); (**h**) H_2_SO_4_ (90 °C)/HCl (70 °C) (10,000× magnification); (**i**) H_2_SO_4_ (120 °C)/HCl (60 °C) (1000× magnification); (**j**) H_2_SO_4_ (90 °C)/HCl (60 °C) (10,000× magnification); (**k**) H_2_SO_4_ (120 °C)/HCl (80 °C) (1000× magnification); and (**l**) H_2_SO_4_ (120 °C)/HCl (80 °C) (10,000× magnification).

To understand the etching effect of the temperature on the surface morphology the surface topography of etching in H_2_SO_4_ at 90–120 °C and HCl at 60–80 °C were tested. We were able to establish a lower etching reaction temperature and an uneven distribution of holes, as shown in Figure 10. In addition, fewer nanoholes were formed after etching at a lower temperature. Finally, acid etching by using H_2_SO_4_ at 120 °C and HCl at 80 °C could yield uniform micrometer-nanometer 3D holes.

#### 3.2.3. Roughness Analysis

The surface roughness of the Ti and Ti-6Al-4V ELI after sandblasting were measured to be 4.28 μm and 4.13 μm, respectively, using white-light interferometry (Table 7 and Table 8). When the CP Ti was etched using H_2_SO_4_ and HCl at BT, the roughness of the CP Ti and the Ti-6Al-4V ELI were 3.62 and 1.47 μm, respectively. When the CP Ti and Ti-6Al-4V ELI were etched using HCl and H_2_SO_4_ at BT, the roughness of CP Ti and Ti-6Al-4V ELI were 2.5 and 1.73 μm, respectively. This produced HF with a higher etching rate for metal and a low level of surface roughness. Therefore, the CP Ti had superior anti-acid characteristics and increased roughness after acid etching.

**Figure 11:**
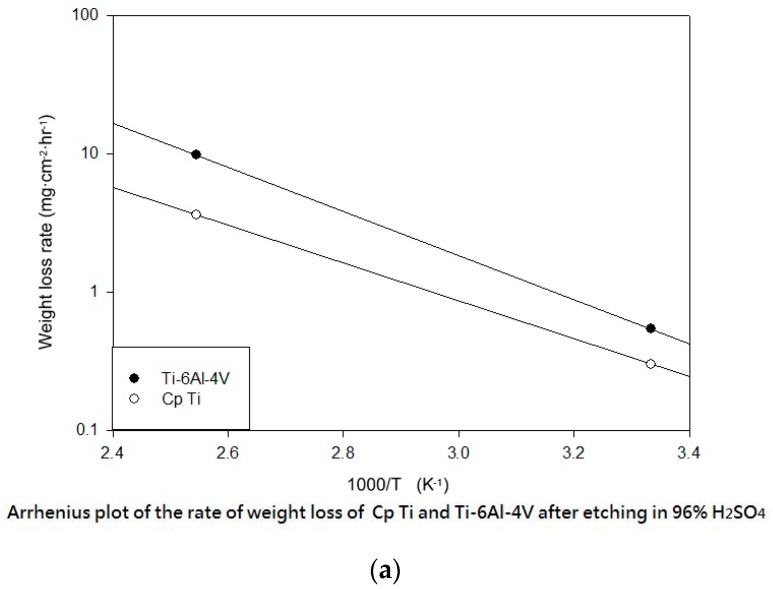

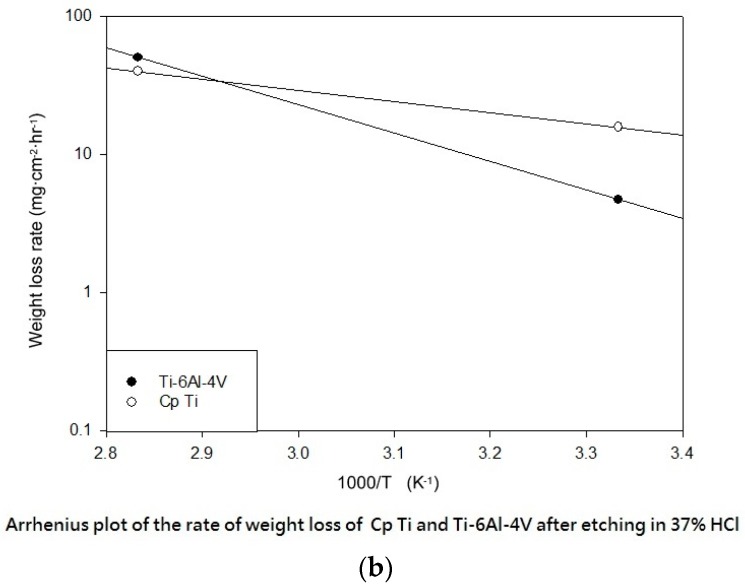
The Arrhenius plot of the etching rate and weight loss of the CP Ti and Ti-6Al-4V after etching in (**a**) H_2_SO_4_; and (**b**) HCl.

Figure 11 shows the Arrhenius plot of the etching rate and weight loss of the CP Ti and Ti-6Al-4V after etching in H_2_SO_4_ and HCl, respectively. According to the mentioned results of the etching temperature, time, and weight loss in our experiments, we could discuss the activation energy of the H_2_SO_4_ and HCl. The activation energy is defined as (−*R*) times the slope of a plot of ln(*k*) vs. (1/T). The activation energy of Ti was 76.51 kJ/mol when etching by using H_2_SO_4_ at BT. The activation energy of Ti-6Al-4V was 88.96 kJ/mol when etching by using H_2_SO_4_ at BT (Figure 11a).

Ti-6Al-4V ELI has a higher level of activation energy than Ti in HCl, which results in higher surface roughness after acid etching (Figure 11b). The activation energy of Ti was 47.53 kJ/mol when etching by using HCl at BT. The activation energy of Ti-6Al-4V was 121.14 kJ/mol when etching by using HCl at BT (Figure 11b).

According to the experimental results, the Arrhenius equation is not suitable for compound (H_2_SO_4_/HCl or HCl/H_2_SO_4_) metal corrosion reactions. This is because these kind of metal acid etching reactions include multiple redox and etching reactions. However, the experimental results can still be inferred, as the titanium and titanium alloy for HCl and H_2_SO_4_ etching rates and surface roughness on the impact of the surface properties of the study should be have contributed.

## 4. Conclusions

This study also successfully explored the process parameters of surface treatment, such as the etching rate, acid concentration, reaction temperature, and reaction time, the optimal values of which can be used to obtain uniform nanopores suitable for bone cell growth. A CP Ti/Ti-6Al-4V ELI surface as tested using shot blasting (pressure, grain size, blasting distance, blasting angle, and time) and acid etching to investigate its topographical characteristics, weight loss, surface roughness, and activation energy. The activation energy of Ti/Ti-6Al-4V ELI after etching using H_2_SO_4_ or HCl at different temperatures was determined. In addition, white-light interferometry was applied to measure the surface nanomorphology of the implant to obtain 2D or 3D roughness parameters. The Ti/Ti-6Al-4V ELI etching rate of the HCl or H_2_SO_4_ at RT was low, whereas HF exhibited the fastest etching rate 9.28 mg/min. After blasting, the acid etching occurred in a short period without initially requiring the etching of the oxide layer in HF. The surface roughness of the Ti, after etching in boiling HCl and H_2_SO_4_, was approximately 1.23 μm. The analysis of the results demonstrated that etching Ti metal in boiling H_2_SO_4_ or HCl aided in the formation of nanoporous structures. Since Ti-6Al-4V is an α + β crystal phase material and its etching rate differs, the variability of the surface roughness was more substantial than that found for Ti. The activation energy of the Ti was 76.51 kJ/mol when etched using H_2_SO_4_ at BT. The activation energy of Ti-6Al-4V was 88.96 kJ/mol when etched using H_2_SO_4_ at BT. This study also indicated that etching with concentrated hydraulic acid results in superior surface modification effects on Ti compared with when H_2_SO_4_ is used.

## Figures and Tables

**Table 1:** The concentration of the acid used in the study.

Etchant	Concentration
HF	1%
H_2_SO_4_	96%
HCl	37%

**Table 2:** The etching parameters for a single type of acid at different temperature.

HF	R.T., (room temperature).
HCl	R.T., Boiling Temp (BT).
H_2_SO_4_	R.T., Boiling Temp.

**Table 3:** The etching parameters for multiple types of acid.

HF 30 s/HCl at boiling/H_2_SO_4_ at boiling
HCl at boiling/H_2_SO_4_ at boiling
H_2_SO_4_ at boiling/HCl at boiling

**Table 4:** The etching rates of Ti-6Al-4V ELI for different etchant solutions at RT or BT.

Etchant	Temp.	Etching Rate (mg/min)
HCl	RT	−0.078
	BT	−0.834
H_2_SO_4_	RT	−0.009
	BT	−0.162

**Table 5:** The etching rates of CP Ti for different etchant solutions at RT or BT.

Etchant	Temp.	Etching Rate (mg/min)
HCl	RT	−0.260
	BT	−0.659
H_2_SO_4_	RT	−0.005
	BT	−0.060

**Table 6:** The difference in weight loss between etching with/without HF treatment.

Etchant	Specimen Weight before Etching (g)	Specimen Weight after Etching (g)	Weight Loss (mg)
HF 30 s	9.367917	9.356587	−11.330
HF 30 s, HCl (80 °C) and H_2_SO_4_ (120 °C)	9.373910	9.270822	−103.088
HCl (80 °C) and H_2_SO_4_ (120 °C)	9.348086	9.254863	−93.223

**Table 7:** The surface roughness of CP Ti after sandblasting, etching in dual acid by using white-light interferometry.

Etchant	Roughness before Etching	Roughness after Etching	Difference of the Roughness
H_2_SO_4_ (120 °C) and HCl (80 °C)	4.28	3.62	−0.66
HCl (80 °C) and H_2_SO_4_ (120 °C)	4.28	2.50	−1.78

**Table 8:** The surface roughness of Ti-6Al-4V ELI after sandblasting and etching in dual acid by using white-light interferometry.

Etchant	Roughness before Etching	Roughness after Etching	Difference of the Roughness
H_2_SO_4_ (120 °C) and HCl (80 °C)	4.13	1.47	−2.66
HCl (80 °C) and H_2_SO_4_ (120 °C)	4.13	1.73	−2.40
